# Loss of Tumor Suppressor C9orf9 Promotes Metastasis in Colorectal Cancer

**DOI:** 10.3390/biom13020312

**Published:** 2023-02-07

**Authors:** Erfei Chen, Fangfang Yang, Qiqi Li, Tong Li, Danni Yao, Lichao Cao, Jin Yang

**Affiliations:** 1 Key Laboratory of Resource Biology and Biotechnology in Western China, Ministry of Education, Northwest University, Xi’an 710069, China; 2 Institute of Preventive Genomic Medicine, School of Life Sciences, Northwest University, Xi’an 710069, China

**Keywords:** colorectal cancer, metastasis, tumor suppressor, epithelial mesenchymal transition

## Abstract

The whole genome sequencing of tumor samples identifies thousands of somatic mutations. However, the function of these genes or mutations in regulating cancer progression remains unclear. We previously performed exome sequencing in patients with colorectal cancer, and identified one splicing mutation in *C9orf9*. The subsequent target sequencing of *C9orf9* gene based on a validation cohort of 50 samples also found two function mutations, indicating that the loss of wild-type C9orf9 may participate in the tumorigenesis of colorectal cancer. In this research, we aimed to further confirm the function of C9orf9 in the CRC phenotype. Our Q-PCR analysis of the tumor and matched normal samples found that C9orf9 was downregulated in the CRC samples. Function assays revealed that C9orf9 exerts its tumor suppressor role mainly on cancer cell migration and invasion, and its loss was essential for certain tumor-microenvironment signals to induce EMT and metastasis in vivo. RNA-sequencing showed that stable-expressing C9orf9 can inhibit the expression of several metastasis-related genes and pathways, including vascular endothelial growth factor A (VEGFA), one of the essential endothelial cell mitogens which plays a critical role in normal physiological and tumor angiogenesis. Overall, our results showed that the loss of C9orf9 contributes to the malignant phenotype of CRC. C9orf9 may serve as a novel metastasis repressor for CRC.

## 1. Introduction

Colorectal cancer (CRC) is the third most common cancers and the second leading cause of cancer-related death worldwide, and its incidence rate has been rising in the past few years [1]. Driver gene mutations and epigenetic alterations play essential roles in CRC initiation and metastasis [2]. Metastasis is a major cause that contributes to the high mortality rate of CRC, and a deeper understanding of the molecular basis of metastasis is of great clinical significance. Genome-wide screening using next-generation-sequencing (NGS) is a powerful approach with the potential to discover the genetic atlas of cancer genomes [3]. Specifically, efforts have been made to identify metastasis-related genes that are responsible for inhibiting the metastasis but not suppressing the growth of primary tumors, such as E-cadherin, NDRG1, and NME1 [4]. Nevertheless, the function of mutated genes with a high penetrance have yet to be explored.

In our recent study, we performed the exome sequencing of unrelated CRC patients, and identified loss-of-function (LoF) mutations in several putative tumor suppressor genes, among which a splicing mutation of *C9orf9* was observed in one CRC patient [5]. The preliminary screening of five digestive tumor samples obtained from public data also revealed that the loss of C9orf9 expression might be CRC-specific, indicating that *C9orf9* could be a new candidate gene for CRC. The *C9orf9* gene is located at the tumor suppressor locus 9q34.1-2, and its mRNA level is significantly reduced in bladder cancer [6]. However, the function of this uncharacterized protein in cancer has not been well illustrated. In this study, to prove our hypothesis that C9orf9 acts as a tumor suppressor in CRC, we performed experiments both in vitro and in vivo. Our work confirmed that C9orf9 is decreased in CRC patients and correlated with metastasis. The presence of wild-type C9orf9 inhibited CRC progression partially via metabolism and EMT-related pathway regulation. In summary, these findings provide new insights into the mechanism of C9orf9-mediated CRC metastasis.

## 2. Results

### 2.1. Deep Sequencing Identified Novel Somatic Mutations of C9orf9 in CRC Samples

**Figure 1:**
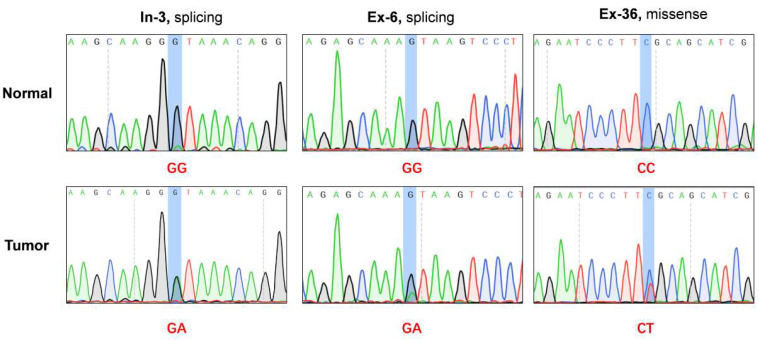
**Exome sequencing and deep sequencing identified three somatic mutations in *C9orf9* gene**. In-3: patient from initial exome sequencing; Ex-6, Ex-36, patients from extended validation cohort. Somatic mutations were visualized and analyzed by SnapGene v4.2.

In our published exome profile, we identified a splice site mutation (Chr9:135754402, G > A) of C9orf9 in one CRC sample (initial exome sequencing sample, In-3) [5]. To avoid obtaining a false positive result, we amplified the corresponding regions of tumor tissue and matched normal control using a PCR assay and carried out Sanger sequencing (Figure 1). To identify novel function mutations, we carried out the target sequencing of the C9orf9 coding region in a validation group of 50 pairs of CRC samples and detected a novel splicing mutation (ISV2 + 1G > A) in the Ex-6 patient and a missense mutation (c.25C > T) in the Ex-36 patient (Table 1). Using Cancer Variant Predictor, a panel of functional analysis through Hidden Markov Models (fathmm) [7], we analyzed three mutations to predict the oncogenic status (disease-driver or neutral) of the somatic point mutations. The oncogenic score for ISV1 + 1G > A, ISV2 + 1G > A, and c. 25C > T were 0.725, 0.975, and 0.619, respectively, indicating that these mutations leading to the loss of wild-type of C9orf9 may affect the oncogenesis of CRC.

### 2.2. The Expression Analysis of C9orf9 in Digestive Tumor Samples

**Figure 2:**
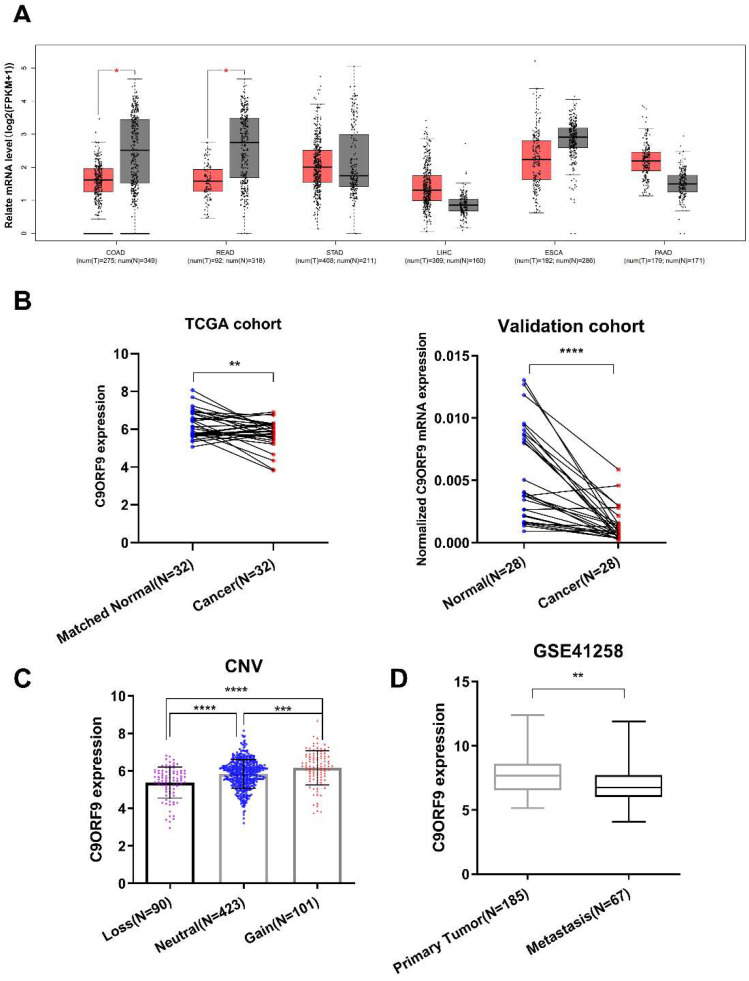
**Gene expression analysis of C9orf9 in multiple samples of digestive tumor patients**. (**A**). Analysis of C9orf9 expression in five digestive tumors from TCGA and GTEx samples. * *p* < 0.05. T: tumor (red), N: normal (grey). (**B**). C9orf9 expression level in tumor and matched normal tissues from TCGA and local validation cohort. ** *p* < 0.01, *** *p* < 0.001. (**C**). Correlation between copy number variation (CNV) and C9orf9 expression level in TCGA cohort. **** *p* < 0.0001. (**D**). C9orf9 is downregulated in metastatic tumor samples of CRC, data from GSE41258. ** *p* < 0.01.

A previous study reported that frequent deletions on 9q34.1-2 were detected in bladder transitional cell carcinoma, and C9orf9 mRNA level was significantly reduced [6]. Here, utilizing TCGA and GTEx data, we examined the C9orf9 expression pattern in five digestive tract system cancer types, including colon and rectum cancer, esophageal cancer, gastric cancer, liver cancer, and pancreatic cancer. We found that C9orf9 was significantly decreased in colon and rectum cancer (*p* < 0.05, Figure 2A) but not in the other tumor types. Furthermore, we detected decreased level of C9orf9 in both the TCGA CRC patients (*n* = 32) and our validation cohort (*n* = 28) (Figure 2B). Next, we analyzed the expression data together with the clinical data of the TCGA samples in order to further determine whether C9orf9 is correlated with CRC progression. We found that C9orf9 expression is highly correlated with the copy number variation (Figure 2C). The χ2 test showed C9orf9 mRNA level is significantly correlated with the patients’ lymphatic invasion but not with age, gender, the tissue site, or presence of colon polyps. We also observed that patients with disease of an advanced stage showed relatively lower expression of C9orf9, although it was not statistically significant (*p* = 0.148, Table 2). We then performed an analysis comparing the primary tumor and metastatic samples from the GSE41258 dataset, and confirmed that C9orf9 expression was downregulated in patients with metastatic CRC (*p* < 0.01, Figure 2D). Taken together, abnormal C9orf9 expression may be associated with the malignant progression of CRC.

### 2.3. C9orf9 Has a Limited Effect on Cell Growth

**Figure 3:**
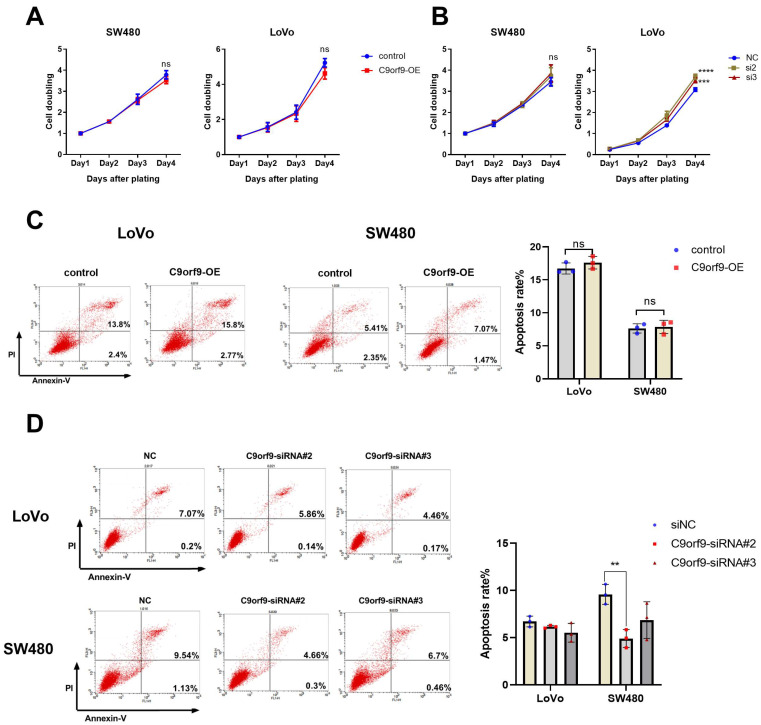
**C9orf9 has limit effect on cell growth.** (**A**). Overexpression of C9orf9 in SW480 and LoVo cells did not affect cell proliferation. (**B**). Knockdown of C9orf9 slightly promoted cell proliferation in LoVo cells, but not in SW480. (**C**). Cell apoptosis analysis using Annexin/PI double staining in LoVo and SW480 cells transfected with C9orf9 expression plasmid. (**D**). Cell apoptosis analysis using Annexin/PI double staining in LoVo and SW480 cells transfected with C9orf9-specific siRNAs. Ns, not significant, ** *p* < 0.01, *** *p* < 0.001, **** *p* < 0.0001.

To elucidate the functional role of C9orf9, we carried out both gain- and loss-of-function assays on two CRC cell lines, namely SW480 and LoVo cells. The knockdown efficiency of the two C9orf9-siRNA was determined by Western blot (Figure S1). First, we utilized the CCK-8 assay to study the cell proliferation, and the difference in C9orf9 overexpression or knockdown groups was not significant as compared to the controls (Figure 3A). In LoVo and SW480 cells, the loss of C9orf9 could slightly promote the cell proliferation ability, although the difference in SW480 was not statistically significant (Figure 3B). Similarly, flow cytometry showed that the gain of C9orf9 did not affect apoptosis, and the apoptosis rate only decreased after knocking down C9orf9 in both cells (Figure 3C,D). In addition, neither gain nor loss of C9orf9 affected cell cycle arrest (Figure S2). These data show that the loss of C9orf9, but not gain of C9orf9, can slightly affect the cell growth capacity, but the effect is limited.

### 2.4. C9orf9 Regulates Cell Migration and Invasion In Vitro and In Vivo

**Figure 4:**
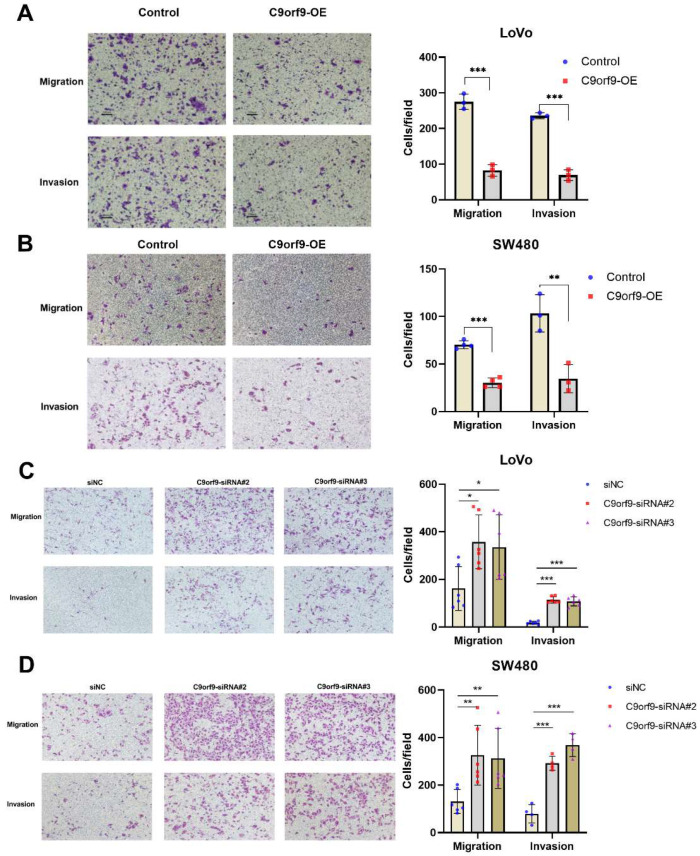
**C9orf9 regulates LoVo and SW480 cell migration and invasion capacity.** Transwell assays (Matrigel-free) or Transwell invasion assays (coated with Matrigel) were performed, respectively, in control and C9orf9-overexpression LoVo (**A**) and SW480 (**B**) cells. (**C**). Cell migration and invasion assays in control and C9orf9-knockdown LoVo cells. (**D**). Cell migration and invasion assays in control and C9orf9-knockdown SW480 cells. * *p* < 0.05, ** *p* < 0.01, *** *p* < 0.001.

Next, we analyzed the effects of both the gain and loss of C9orf9 on CRC cell migration and invasion. As shown in Figure 4A, B, utilizing a Transwell assay, C9orf9 weakened the LoVo and SW480 cell migration ability as compared to the controls. Conversely, inhibition of C9orf9 in both cells enhanced the migration capacity. To confirm the results, we also performed another method, namely wound scratch assays (Figures S3 and S4). Specially, the cell migration ability was markedly enhanced after knocking-down C9orf9 in both cells. In addition, we used the Matrigel invasion assay to explore the effect of C9orf9 on cancer cell invasion. The consistent result showed that a high level of C9orf9 can inhibit CRC cell invasion (Figure 4A–D).

**Figure 5:**
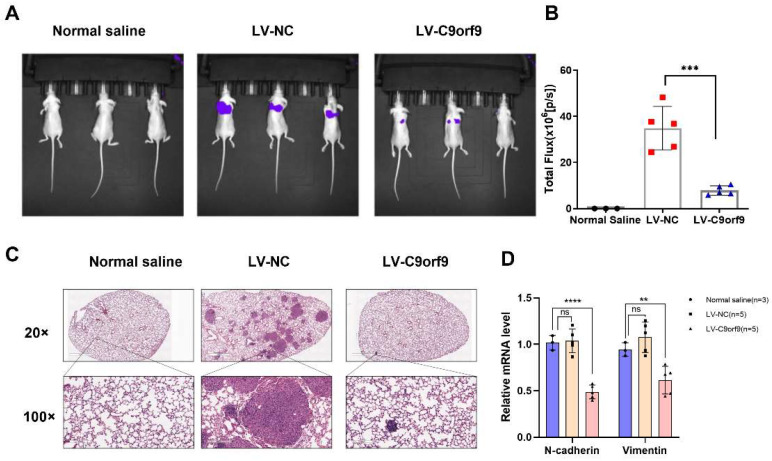
**C9orf9 inhibits tumor metastasis in vivo.** (**A**). Representative images of luciferase expression from lung metastasis of the normal saline (*n* = 3), LV-NC (*n* = 5), and LV-C9orf9 (*n* = 5) groups. (**B**). Quantification of the total flux, compared with the LV-NC group. (**C**). HE staining of lungs from the normal saline, LV-NC, and LV-C9orf9 groups. (**D**). mRNA level of metastasis-related marker N-cadherin and Vimentin. ** *p* < 0.01, *** *p* < 0.001, **** *p* < 0.0001.

To determine the tumor suppressor effect of C9orf9 on CRC metastasis in vivo, we used the metastatic cell line LoVo to employ a tail vein injection metastasis model. We constructed C9orf9 stable-expressing LoVo cells by lentivirus infection. A control saline, LV-NC, and LV-C9orf9 were injected into nude mice through the tail vein. The luciferase activity in the lungs of C9orf9 overexpression group was significantly lower than that of the LV-NC group (Figure 5A,B). Compared with the NC group, there were fewer intrapulmonary metastases in the C9orf9 overexpression group (Figure 5C). Additionally, we detected the downregulation of the metastasis-related markers N-cadherin and Vimentin in the tumors from the LV-C9orf9 group (Figure 5D). Since our in vitro results showed that the overexpression of C9orf9 did not change the proliferation or survival phenotype (Figure 3A,C), this could be a major result of metastasis. The abovementioned data suggest that C9orf9 regulates the migration and invasion capacities of CRC cells both in vitro and in vivo.

### 2.5. C9orf9 Responds to Various Tumor Microenvironment Factors and Modulates Epithelial Mesenchymal Transition in CRC Cells

**Figure 6:**
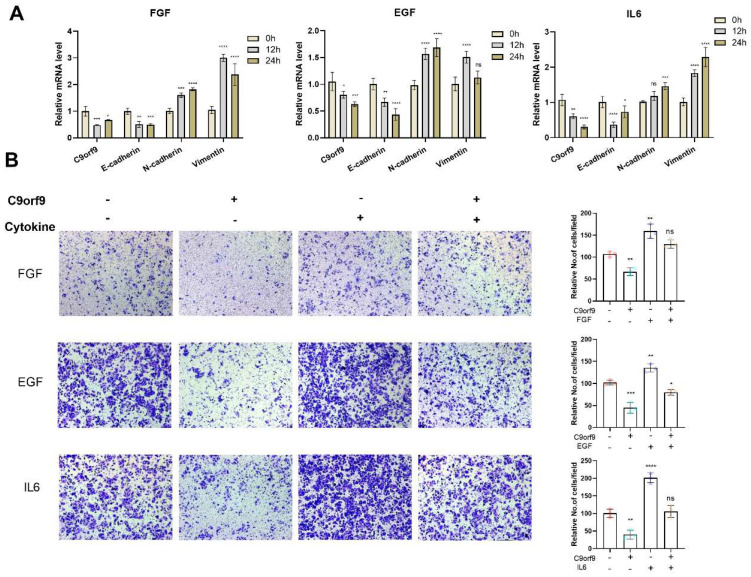
**C9orf9 responds to and involves in FGF, EGF, and IL6 induced EMT.** (**A**). Q-PCR analysis of C9orf9 and EMT-related markers (E-cadherin, N-cadherin, Vimentin) in FGF (20 ng/mL), EGF (20 ng/mL), and IL6 (50 ng/mL)-stimulated SW480 cells at 0, 12, and 24 h. (**B**) Transwell (Matrigel-free) assays of LV-NC and LV-C9orf9 SW480 cells with or without cytokine stimulation. * *p* < 0.05, ** *p* < 0.01, *** *p* < 0.001, **** *p* < 0.0001.

An increasing wealth of evidence has linked epithelial mesenchymal transition (EMT) with the malignant phenomenon of cancer metastasis. EMT is considered as a specific cellular response to various microenvironments. Therefore, we aimed to determine whether C9orf9 is involved in the microenvironment-factors-induced EMT. In the epithelial SW480 cells, C9orf9 could be inhibited by multiple previously reported EMT inducers, including FGF, EGF, and IL-6 (Figure 6A), but not by TGF-β and IGF (Figure S5). The expression of the epithelial marker E-cadherin decreased, while the mesenchymal markers N-cadherin and Vimentin increased, indicating that the cytokine-induced EMT model is robust. We also noticed that the addition of TGF-β or IGF did not change the mRNA level of C9orf9.

To determine the dynamic change in C9orf9 in microenvironment-induced EMT, we utilized the Transwell assay to evaluate the migratory capacity in cytokine-induced wild-type SW480 or C9orf9-stable-overexpressing SW480 cells. Consistently, the addition of FGF, EGF, or IL-6 enhanced SW480 cell migration, while the overexpression of C9orf9 rescued the malignant phenotype (Figure 6B). These data suggest that C9orf9 may play certain roles in the EMT process.

### 2.6. C9orf9 Regulates Metastasis-Related Genes and Pathways

**Figure 7:**
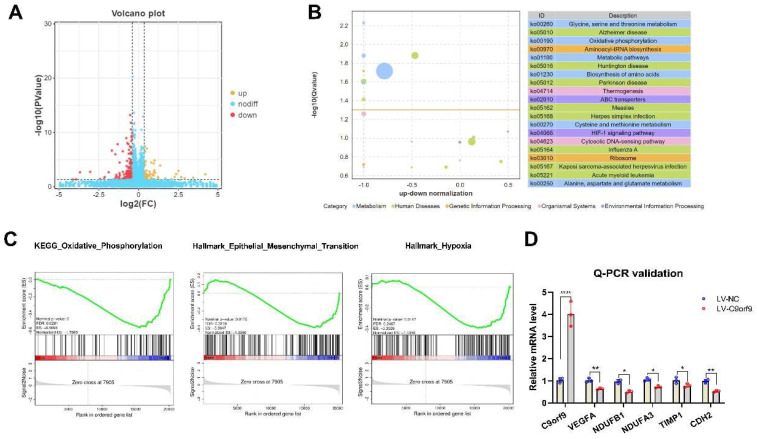
**C9orf9 regulates metastasis-related gene and pathway.** (**A**). Volcano plot of differentially expressed genes (DEGs). (**B**). Top 20 KEGG pathway enrichment from DEGs. (**C**). GSEA plot of oxidative phosphorylation, EMT, and hypoxia gene set. (**D**). Q-PCR validation of angiogenesis, oxidative phosphorylation, and EMT related genes. * *p* < 0.05, ** *p* < 0.01, **** *p* < 0.0001.

To reveal the role of C9orf9 in CRC, we performed RNA-seq to identify the downstream genes and pathways regulated by C9orf9. Differentially expressed genes (DEGs) between the LV-NC and LV-C9orf9 LoVo cells are shown in Figure 7A. In total, 69 genes were up-regulated and 184 were down-regulated (|FC| ≥ 1.5, *p* < 0.05). Surprisingly, we found that most DEGs were enriched in the metabolism pathway and oxidative phosphorylation (OXPHOS) pathway (Figure 7B). Considering that C9orf9 greatly influences migration, invasion, and the EMT phenotype, we then focused on the metastasis-related gene sets through Gene Set Enrichment Analysis (GSEA). The genes in the OXPHOS, EMT, and hypoxia sets were significantly enriched and highly expressed in the control groups (Nominal *p* < 0.05, Figure 7C). We selected significant DEGs for QPCR validation, among which VEGFA (angiogenesis-related), TIMP1, CDH2 (EMT-related), and NDUFs (OXPHOS-related) were consistent with the RNA-seq results.

Consequently, together with the gene expression analysis, cell phenotype assays, and RNA-seq analysis, we confirmed that C9orf9 plays a tumor suppressor role via the regulation of metastasis-related signaling pathways.

## 3. Discussion

Exome sequencing is a cost-effective method that can detect rare mutations in a high-throughput manner. The whole exome sequencing of cancer patients can help us to understand the potential biological pathogenesis of certain cancers. Our recent work revealed that loss-of-function mutation screening is a powerful strategy that can be employed to identify novel tumor suppressor genes, and we dissected the function of several genes in the CRC model [5,8,9]. In this study, we reported that C9orf9 participates in the progression of CRC. C9orf9 was first reported to be correlated with bladder cancer [6]. Boelens et al. also found that C9orf9 expression was down-regulated in squamous cell lung cancer compared to normal epithelium in smokers [10]. However, to date, the mutation and expression profile of C9orf9 in digestive tumors and its underlying function in cancer have not been comprehensively investigated.

We identified two splicing mutations and one missense mutation in CRC patients, and all mutations were predicted to be oncogenic. Genome wide/transcriptome sequencing studies have shown that the regulation of splicing is complex and that it occurs in cooperative transcription and is influenced by chromatin status and mRNA modification [11]. Many of the molecular changes observed in cancer result from modifications in the splicing process, including mutations in splicing body proteins, mutations in pre mRNA regulatory sequences, and changes in the expression of splicing regulatory factors. These changes may eventually lead to disorders in cell differentiation, survival, and invasion [12]. To fully unravel the roles of C9orf9 splicing mutations in CRC, based on our research, further experiments are required to elucidate the underlying mechanism, including CRISPR/Cas9-based point mutation in CRC cells.

Here, we analyzed the expression of C9orf9 in five digestive tumors using TCGA and GTEx data. Significant differences were found only in colon and rectum cancer, and we observed that the low expression of C9orf9 is correlated with metastasis and an advanced tumor stage. These results indicated the tumor suppressor role of C9orf9 might be colon tissue-specific. To further confirm our hypothesis that the loss of wild-type C9orf9 might be involved in the pathogenesis of CRC, we performed systematic experiments both in vitro and in vivo. Our results consistently revealed that C9orf9 functions mainly in cell migration, metastasis, and EMT.

Metastasis is a hallmark of cancer. Cells from primary tumors invade other parts of the body and form new tumors. It is the main cause of death among more than 90% of cancer patients [13]. In CRC patients, metastasis mainly involves the liver and lung [14]. Some studies have found that mutations in key driving genes (KRAS, p53, SMAD4) are related to CRC metastasis [15]. Our results revealed that C9orf9 can not only inhibit cell migration and invasion, but also modulate the EMT phenotype of CRC cells. Moreover, C9orf9 responds to well-established EGF, FGF, and IL-6 induced EMT, indicating the loss of C9orf9 is involved in the cytokine-signal-triggered EMT process. EMT is involved and plays a key role in cancer cell metastasis, as it transforms epithelial cells into mesenchymal cells to promote metastasis [16]. Through our RNA-seq data, we identified several angiogenesis, EMT, and OXPHOS related genes. The angiogenesis marker VEGFA is significantly reduced when overexpressing C9orf9, suggesting that C9orf9 could potentially oppose the HIF1-α/VEGF pathways. VEGFA related signal transduction plays a crucial role in the migration of cancer cells from their primary niche to their secondary sites [17]. Therefore, identifying the mechanisms or drugs that inhibit VEGF related pathway members may provide a means of reducing the incidence of distant metastasis. Although our RNA-seq results demonstrated that the DEGs are not enriched in the canonical Wnt, PI3K, or MAPK pathways, they are mainly enriched in metabolism-related pathways. It is well-known that cancer cells alter their metabolic profiles during tumorigenesis and metastasis, thus displaying a tightly regulated metabolic plasticity program [18]. Overall, our data emphasize the key role of C9orf9 in CRC metastasis.

In summary, in the present study, we revealed that C9orf9 may act as a tumor suppressor in regulating CRC EMT and metastasis. Our results shed light on the specific mechanisms of CRC metastasis and treatment.

## 4. Materials and Methods

### 4.1. Patients and Ethics Statement

The tumor and matched normal tissue samples were collected from patients and kindly provided by the Fourth Military Medical University (50 cases for deep sequencing, and 28 cases for Q-PCR validation). All patients provided written informed consent, and this study was approved by the Ethics Committee of Northwest University.

### 4.2. Deep Sequencing of C9orf9 Gene Coding Region

In 50 cases of deep sequencing, we used the QIAamp DNA FFPE Tissue Kit (QIAGEN, Hilden, Germany) according to the instructions to extract both tumor and normal tissue DNA. The exon and exon–intron junction regions of C9orf9 were subsequently amplified using HiFi™ Hot Start (KAPA Biosystems, Cambridge, MA, USA) and Sanger sequenced (the amplification and sequencing primers are listed in Table S1). Somatic mutations were visualized and analyzed by SnapGene v4.2.

### 4.3. TCGA Data Access

From the UCSC Xena database, we downloaded the clinical and gene expression data of five digestive tumor types. The expression data were normalized to log_2_(FPKM + 1).

### 4.4. Cell Lines

Colon epithelial SW480 cells and metastatic LoVo cells were purchased from Procell (Wuhan, China) and cultured in RPMI 1640 medium supplemented with 10% fetal bovine serum (Gibco, Gaithersburg, MD, USA). Cells were cultured in an incubator at 37 °C, 5% CO_2_.

### 4.5. RNA Extraction and Quantitative Real Time PCR

Total RNA was extracted with TRIzol reagent (#9108, Takara, Osaka, Japan). The mRNA was then reverse transcribed to cDNA according to the HiScript RT SuperMix manual for qPCR (#R223, Vazyme, Nanjing, China). Quantitative real-time PCR was performed with the ChamQ Universal SYBR qPCR Master Mix (#Q711, Vazyme, Nanjing, China), and detected by QuantStudio 3 (Applied biosystems, Waltham, MA, USA). The relative expression was normalized according to the formulas 2^−ΔΔCt^, and GAPDH was set as an internal control. The specific primers for the target genes and reference are listed in Table S2.

### 4.6. Western Blot

Protein extraction and quantification were carried out according to a previously described protocol [9]. The antibodies used in this study were as follows: rabbit anti-C9orf9 (#HPA022243, Sigma-Aldrich, St. Louis, MO, USA), mouse anti-GAPDH (#YM3029, ImmunoWay, Beijing, China), and corresponding secondary antibody (goat anti-rabbit #A3687, goat anti-mouse #A3562, Sigma-Aldrich, St. Louis, MO, USA).

### 4.7. Plasmid and siRNA Transfection

The full-length CDS of C9orf9 was amplified using HEK293T cDNA and cloned into the BamHI/SalI sites of the pEF-BOS-EX vector. A total of 2 ug of recombinant plasmid was transfected in a 6-well plate with 4 uL of Lipofactmine3000 (ThermoFisher, Waltham, MA, USA) at a cell confluency of 60–70%. To inhibit C9orf9 expression, two C9orf9-specific siRNAs were synthesized and purified (Tsingke Biotechnology, Beijing, China). The target sequences of siRNA are CAUCCUAGACUUAAUGAAA (C9orf9-siRNA#2) and AGAGCUACAUGGAACACUA (C9orf9-siRNA#3), respectively. SW480 and LoVo Cells were reverse transfected with HiPerFect transfection reagent (#301705, Qiagen, Hilden, Germany) at a concentration of 30 nM according to the manuals.

### 4.8. Lentiviral Infection

For the stable overexpression of C9orf9, the full length CDS was inserted into a pLV17 vector (Luc tag). Lentiviral stocks were prepared in HEK-293T cells. The LoVo or SW480 cells were infected with 5 μg/mL polybrene mixed viral supernatant for 48 h. Then, puromycin (2 μg/mL) was used to screen the cells with a stable overexpression of C9orf9.

### 4.9. Cell Proliferation Assay

Cell Counting Kit-8 (#Ck04, Dojindo Laboratories, Japan) was used to detect the cell viability [19]. Briefly, a total of 5 × 10^3^ SW480 cells or 3 × 10^3^ LoVo cells were seeded onto 96-well plates with 5 replicates. At the indicated time, 10 μL of CCK-8 solution was added to each well. After incubation for 3 h, the viable cells were measured at a wavelength of 450 nm.

### 4.10. Flow Cytometry

Apoptosis was detected using an Annexin V/PI apoptosis assay kit (#A003, 7sea biotech, Shanghai, China) according to the protocol. The early and late apoptotic cells were analyzed using the FACSCalibur flow cytometer (BD Bioscience, San Jose, CA, USA). The method used for the cell cycle assay was described previously [5].

### 4.11. Migration and Invasion Assay

We applied the Transwell chambers (#3422, Corning, New York, NY, USA) to detect cell migration capacity. An amount of 2 × 10^4^ (LoVo) or 5 × 10^4^ (SW480) treated or untreated cells in a total volume of 100 μL serum-free medium were plated in the upper chamber, while 700 μL of complete medium in the bottom chamber. The cells were then incubated at 37 °C for 24 h (LoVo) or 48 h (SW480). The cells were fixed with 4% paraformaldehyde, stained with 0.1% crystal violet, and rinsed with PBS. Similarly, we analyzed the invasion capacity using Transwell coated with 50 μL of Matrigel (#356234, BD Bioscience, San Jose, CA, USA). The migration/invasion capacities were analyzed by counting the number of cells in five randomly picked fields, and three independent experiments were conducted. To further confirm the results, a wound scratch test was also performed to assess the migration ability, and detail method was described as previously [20].

### 4.12. Animal Model

Six-weeks old BALB/c nude mice were randomly divided into normal saline (*n* = 3), LV-NC (*n* = 5), and LV-C9orf9 groups (*n* = 5). A total of 2.0 × 10^6^ LoVo cells were suspended in normal saline and injected through the tail vein. Every week, the mice were intraperitoneally injected with d-luciferin (75 mg/kg) and photographed within 30 min (Luminometer, Roper Scientific). After five weeks, the mice were sacrificed and the lungs were stained with hematoxylin-eosin (HE). All the animal studies were approved by the Animal Care Ethics Committee of Northwest University and performed in accordance with the institutional guidelines.

### 4.13. Library Preparation and Transcriptome Sequencing

Total RNA was extracted using MiniBEST Universal RNA Extraction Kit (#9767, Takara, Osaka, Japan). RNA concentration was evaluated by Qubit (Invitrogen, Carlsbad, CA, USA). The polyA + mRNA was enriched by NEBNext Poly(A) mRNA Magnetic Isolation Module (#E7490L, NEB, Ipswich, MA, USA). Then, the enriched mRNA was reverse transcribed into cDNA with SMARTScribe Reverse Transcriptase (#639537, Clontech, Osaka, Japan). The purified cDNA fragments were subjected to end repair, A-tailing, and Illumina adaptor ligation. The products were size selected (200–500 bp) and PCR amplified for Next-generation sequencing using Illumina Novaseq6000 by Gene Denovo Biotechnology Co (Guangzhou, China).

### 4.14. Statistical Analyses

GraphPad Prism v8 (GraphPad Software, Inc.) was used for statistical analysis. All experiments were repeated at least three times and the results were presented as the mean ± SD. Statistical analyses were performed by Student′s *t* tests or ANOVA test. A *p* value < 0.05 was considered statistically significant.

## Figures and Tables

**Table 1:** Novel somatic mutation of *C9orf9* in CRC samples.

Sample	Mut. Type	Exon/Intron	Location	Base Change	Effect	Oncogenic Score
In-3	Splicing mutation	Intron 1	chr9:135754402	ISV1 + 1G > A	splicing	0.725
Ex-6	Splicing mutation	Intron 2	chr9:135762959	ISV2 + 1G > A	splicing	0.975
Ex-36	Missense	Exon 2	chr9:135758002	c.25C > T	p.Arg9Cys	0.619

**Table 2:** Correlation of C9orf9 expression to clinical variables of TCGA CRC samples.

Variables	Cases	C9orf9 Expression		χ^2^	*p* Value
		High	Low		
**Age (years)**					
>65	380	186	194	0.416	0.519
≤65	258	133	125		
					
**Gender**					
Male	337	164	173	0.574	0.448
Female	298	154	144		
					
**Tissue site**					
Colon	471	237	234	0.073	0.787
Rectum	167	82	85		
					
**Colon polyps present**					
Yes	96	44	52	0.463	0.496
No	218	109	109		
					
**Lymphatic invasion**					
Yes	231	102	129	4.241	0.039 *
No	342	181	161		
					
**Stage**					
I + II	312	167	145	2.092	0.148
III + IV	151	70	81		

* denotes *p* < 0.05.

## Data Availability

The RNA-seq data used in this study have been deposited in the National Center for Biotechnology Information’s Sequence Read Archive (Sequence Read Archive study accession code PRJNA797540).

## Supplementary Materials

The following supporting information can be downloaded at: https://www.mdpi.com/article/10.3390/biom13020312/s1, Figure S1: The knockdown efficiency of two siRNA targeting C9orf9; Figure S2: Cell cycle analysis of C9orf9 overexpression (A) and knockdown (B) in LoVo and SW480 cells; Figure S3: Wound scratch assay in C9orf9 overexpression (A) and knockdown (B) LoVo cells; Figure S4: Wound scratch assay in C9orf9 overexpression (A) and knockdown (B) SW480 cells; Figure S5: C9orf9 did not response to TGF-β (A) or IGF (B)-induced EMT in SW480 cells; Table S1: Amplification and sequencing primers for C9orf9; Table S2: Q-PCR primers used in this study.

## Abbreviations

C9orf9	Chromosome 9 Open Reading Frame 9
CCK-8	cell counting kit-8
CRC	colorectal cancer
EMT	epithelial-mesenchymal transition
GSEA	Gene set enrichment analysis
GTEx	Genotype-Tissue Expression
LoF	Loss-of-Function
NGS	Next Generation Sequencing
OXPHOS	oxidative phosphorylation
TCGA	The Cancer Genome Atlas
