# Supplementary material for: Loss of Tumor Suppressor C9orf9 Promotes Metastasis in Colorectal Cancer
Source: Biomolecules. 2023 Feb 7;13(2):312. doi: 10.3390/biom13020312 (PMC9953698; doi:10.3390/biom13020312)
Supplement: Supplementary file 1 [file biomolecules-13-00312-s001.zip › biomolecules-2123741-supplementary.pdf]

biomolecules

MDPI

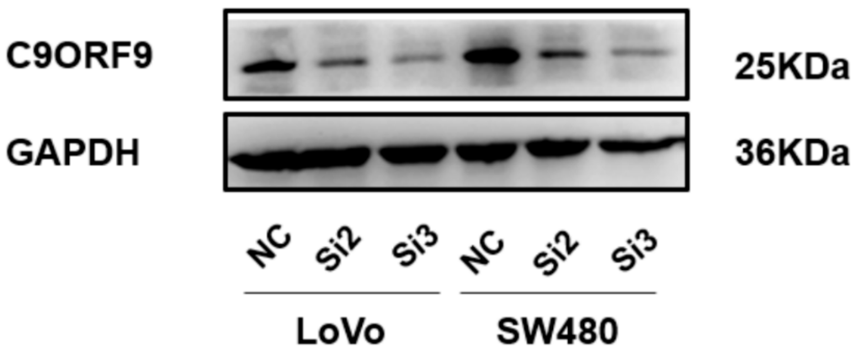

Figure S1. The knockdown efficiency of two siRNA targeting C9orf9.

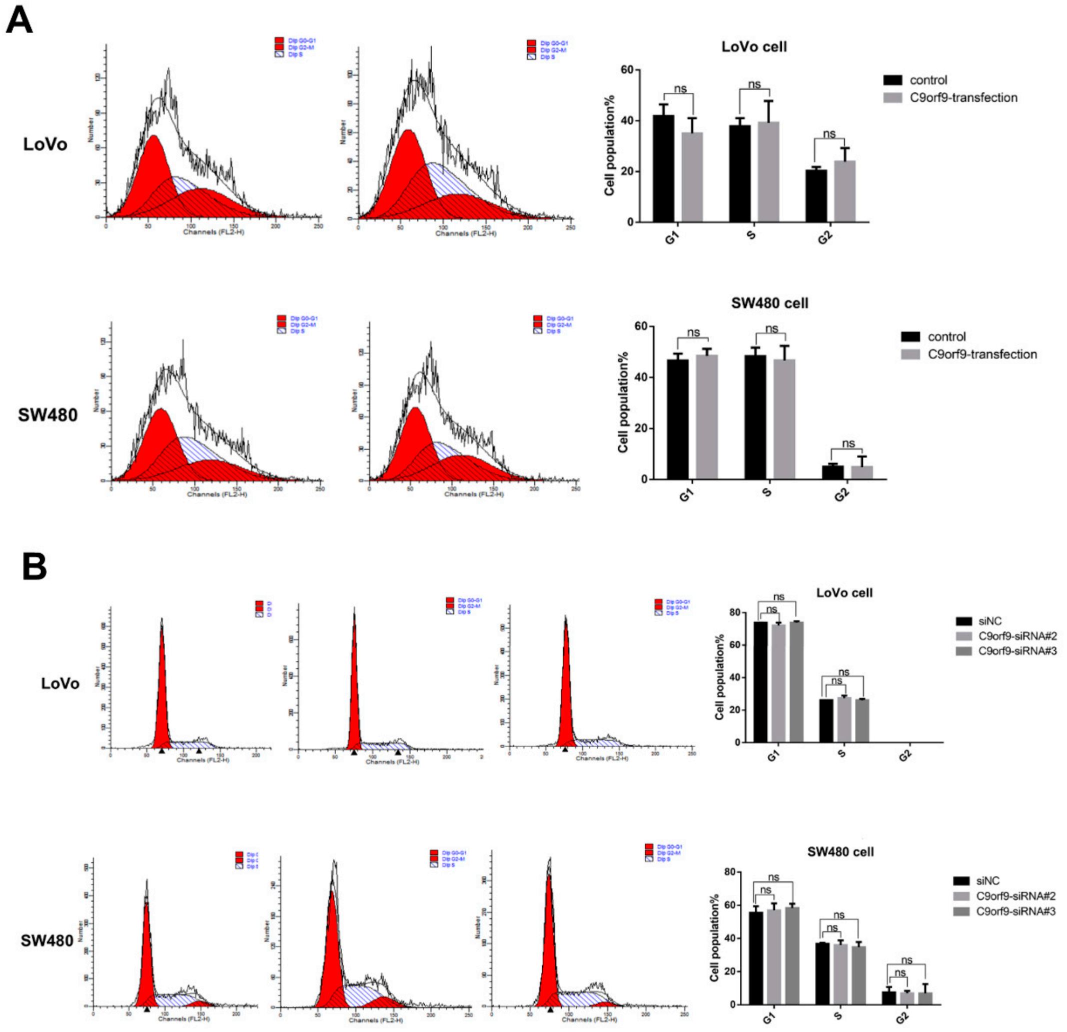

Figure S2. Cell cycle analysis of C9orf9 overexpression (**A**) and knockdown (**B**) in LoVo and SW480 cells.

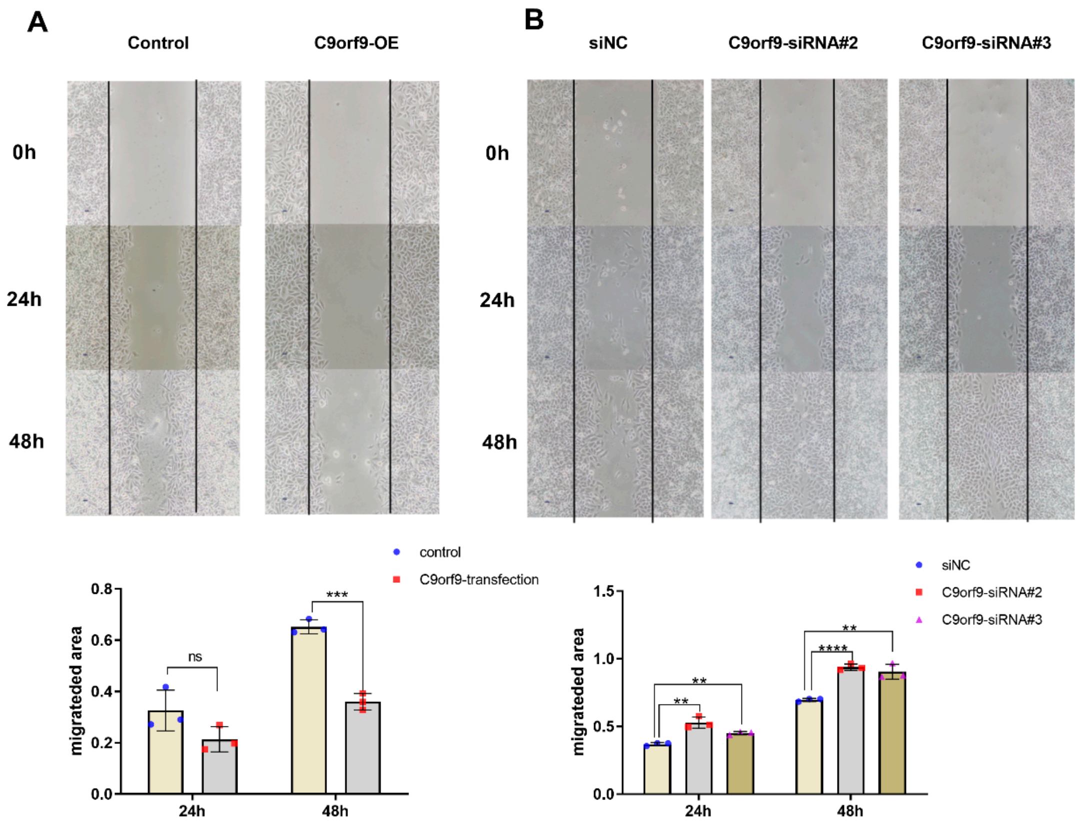

Figure S3. Wound scratch assay in C9orf9 overexpression (**A**) and knockdown (**B**) LoVo cells. $^{**}p < 0.01$, $^{***}p < 0.001$, $^{****}p < 0.0001$.

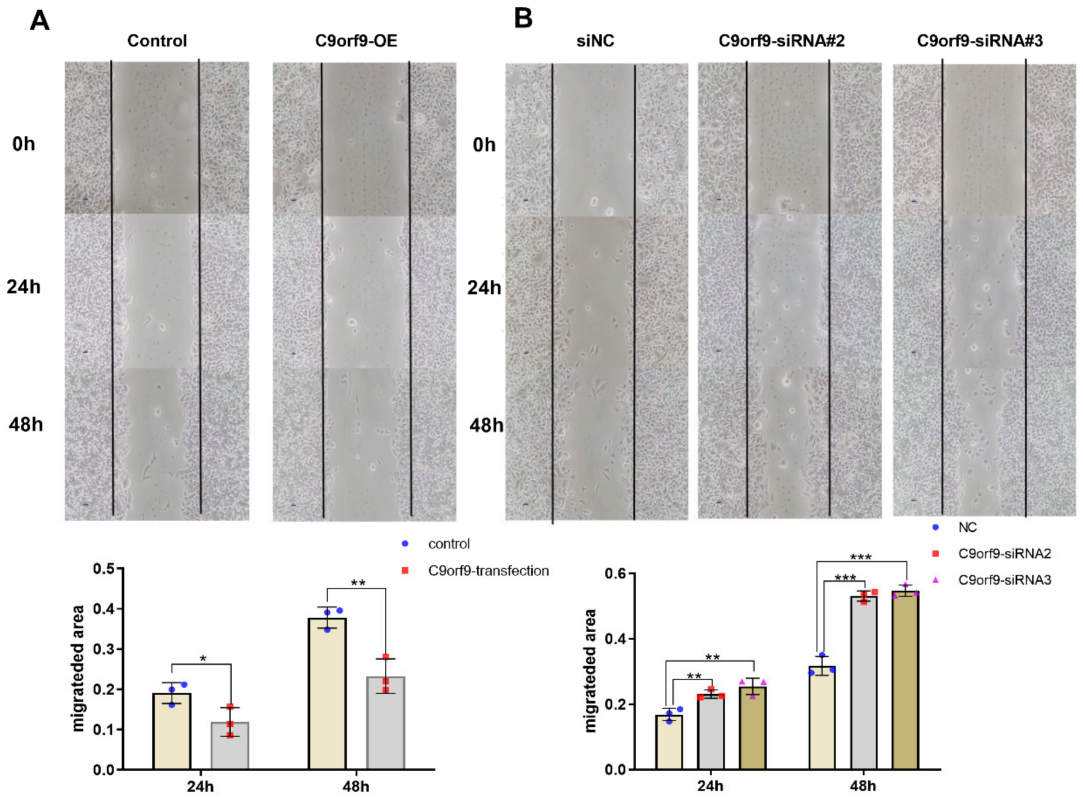

Figure S4. Wound scratch assay in C9orf9 overexpression (**A**) and knockdown (**B**) SW480 cells. $*p < 0.05$, $**p < 0.01$, $***p < 0.001$.

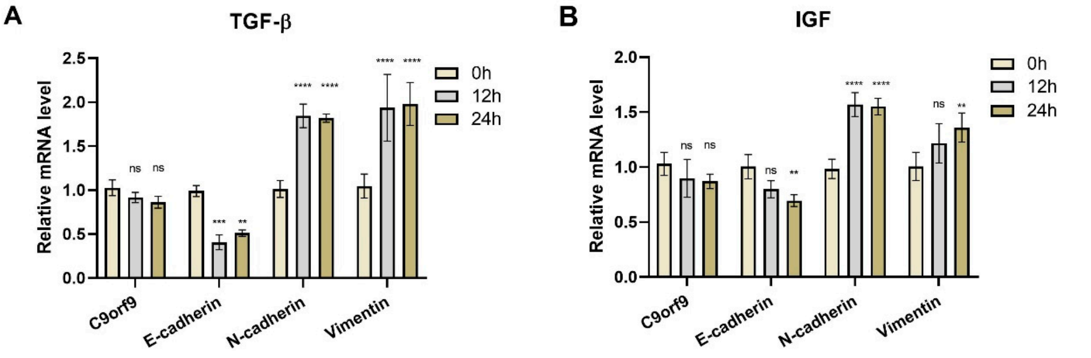

Figure S5. C9orf9 did not response to TGF-β (**A**) or IGF (**B**)-induced EMT in SW480 cells. ns not significant, $**p < 0.01$, $***p < 0.001$, $****p < 0.0001$.

Table S1. Amplification and sequencing primers for C9orf9.

Gene	Exon	5' Primer name	*Sequence*	3' Primer name	*Sequence*	Seq Primer	*Sequence*	PCR Length
C9orf9	E2	>C9orf9_E2_F	AAGGCGCTCCAGTGAGAGGG	>C9orf9_E2_R	CTTTTGTGAGAGGGGACTGG	>C9orf9_E2_F	CCCTGACAGTCCTTCCCTCC	359bp
	E3	>C9orf9_E3_F	GTAGGGTGGGAGGGAGTAGG	>C9orf9_E3_R	GAGCAGCATCAGAGGGACTT	>C9orf9_E3_SF	TGGACATTTGCACCATAAGC	330bp
	E4	>C9orf9_E4_F	GGAGCATGGGTTCACCTG	>C9orf9_E4_SR	GTTTGAGGCTGGCTTTTGTG	>C9orf9_E4_SR3	TGTCTACACTTGTGTTTAGT	309bp
	E5	>C9orf9_E5_F	AAATGTGTCCCTGGCTTCTG	>C9orf9_E5_R	ACTGCCCAGTGTCCTTTGAT	>C9orf9_E5_SR	GTCTTAGGGGAGATGTAGGT	222bp

Table S2. Q-PCR primers used in this study.

Gene	Product length	Forward primer	Reverse primer
CDH1	119 bp	CGAGAGCTACACGTTCACGG	GGGTGTCGAGGGAAAAATAGG
GAPDH	86bp	TGGAAATCCCATCACCATCT	TGGACTCCACGACGTACTCA
C9orf9	111bp	GAGAACGCCCACGACAAGA	CAGGAACATGAGCAGAACCC
CDH2	123bp	TGCGGTACAGTGTAACTGGG	GAAACCGGGCTATCTGCTCG
VIM	98bp	AGTCCACTGAGTACCGGAGAC	CATTTCACGCATCTGGCGTTC
TIMP1	96bp	ACCACCTTATACCAGCGTTATGA	GGTGTAGACGAACCGGATGTC
VEGFA	75bp	AGGGCAGAATCATCACGAAGT	AGGGTCTCGATTGGATGGCA
NDUFB1	61bp	GTCCCTATGGGATTTGTCATTGG	CAGTTAGCCGTTCATCACTCTT
NDUFA3	95bp	GGGGCCTCGCTGTAATTCTG	GACGGGCACTGGGTAGTTG
